# Evaluating Cellularity Estimation Methods: Comparing AI Counting with Pathologists’ Visual Estimates

**DOI:** 10.3390/diagnostics14111115

**Published:** 2024-05-28

**Authors:** Tomoharu Kiyuna, Eric Cosatto, Kanako C. Hatanaka, Tomoyuki Yokose, Koji Tsuta, Noriko Motoi, Keishi Makita, Ai Shimizu, Toshiya Shinohara, Akira Suzuki, Emi Takakuwa, Yasunari Takakuwa, Takahiro Tsuji, Mitsuhiro Tsujiwaki, Mitsuru Yanai, Sayaka Yuzawa, Maki Ogura, Yutaka Hatanaka

**Affiliations:** 1 Healthcare Life Science Division, NEC Corporation, Tokyo 108-8556, Japan; maki.ogura@nec.com; 2 Department of Machine Learning, NEC Laboratories America, Princeton, NJ 08540, USA; cosatto@nec-labs.com; 3 Center for Development of Advanced Diagnostics (C-DAD), Hokkaido University Hospital, Sapporo 060-8648, Japan; yhatanaka@huhp.hokudai.ac.jp; 4 Department of Pathology, Kanagawa Cancer Center, Yokohama 241-8515, Japan; kamaboko8ty@gmail.com; 5 Department of Pathology, Kansai Medical University, Osaka 573-1010, Japan; tsutakoj@hirakata.kmu.ac.jp; 6 Department of Pathology, Saitama Cancer Center, Saitama 362-0806, Japan; motoi.noriko@saitama-pho.jp; 7 Department of Pathology, Oji General Hospital, Tomakomai 053-8506, Japan; 8 Department of Surgical Pathology, Hokkaido University Hospital, Sapporo 060-8648, Japan; aishimizu@huhp.hokudai.ac.jp (A.S.); emitaka@huhp.hokudai.ac.jp (E.T.); 9 Department of Pathology, Teine Keijinkai Hospital, Sapporo 006-0811, Japan; 10 Department of Pathology, KKR Sapporo Medical Center, Sapporo 062-0931, Japan; 11 Department of Pathology, NTT Medical Center Sapporo, Sapporo 060-0061, Japan; yasunari.takakuwa@east.ntt.co.jp; 12 Department of Pathology, Sapporo City General Hospital, Sapporo 060-8604, Japan; tsuji.takahiro@gmail.com; 13 Department of Surgical Pathology, Sapporo Medical University Hospital, Sapporo 060-8543, Japan; tsujiwaki@sapmed.ac.jp; 14 Department of Pathology, Sapporo Tokushukai Hospital, Sapporo 004-0041, Japan; mitsuruyanai.md@gmail.com; 15 Department of Diagnostic Pathology, Asahikawa Medical University Hospital, Asahikawa 078-8510, Japan

**Keywords:** tumor content ratio (TCR), next generation sequencing (NGS), artificial intelligence (AI), U-Net model, domain shift, site dependency

## Abstract

The development of next-generation sequencing (NGS) has enabled the discovery of cancer-specific driver gene alternations, making precision medicine possible. However, accurate genetic testing requires a sufficient amount of tumor cells in the specimen. The evaluation of tumor content ratio (TCR) from hematoxylin and eosin (H&E)-stained images has been found to vary between pathologists, making it an important challenge to obtain an accurate TCR. In this study, three pathologists exhaustively labeled all cells in 41 regions from 41 lung cancer cases as either tumor, non-tumor or indistinguishable, thus establishing a “gold standard” TCR. We then compared the accuracy of the TCR estimated by 13 pathologists based on visual assessment and the TCR calculated by an AI model that we have developed. It is a compact and fast model that follows a fully convolutional neural network architecture and produces cell detection maps which can be efficiently post-processed to obtain tumor and non-tumor cell counts from which TCR is calculated. Its raw cell detection accuracy is 92% while its classification accuracy is 84%. The results show that the error between the gold standard TCR and the AI calculation was significantly smaller than that between the gold standard TCR and the pathologist’s visual assessment (p<0.05). Additionally, the robustness of AI models across institutions is a key issue and we demonstrate that the variation in AI was smaller than that in the average of pathologists when evaluated by institution. These findings suggest that the accuracy of tumor cellularity assessments in clinical workflows is significantly improved by the introduction of robust AI models, leading to more efficient genetic testing and ultimately to better patient outcomes.

## 1. Introduction

Genetic panel testing is a fundamental tool for diagnosis and treatment selection in cancer medicine. Since genetic panel testing analyzes the DNA contained in individual cells, accurate sample collection is essential for its success, and a sample with a higher number of tumor cells is considered to be a better specimen. For example, in FDA-approved FoundationOne [1] and OncomineDx Target Test [2], the overall cellularity, or tumor content ratio (TCR) should be more than 20%. For tissues where the overall TCR is between 10% and 20%, micro-dissection and enrichment should be performed to bring up the overall ratio.

In a typical clinical workflow, TCR is estimated manually by pathologists examining H&E-stained slides and is a subjective and error-prone process. Several factors affect the accuracy of counting cells by pathologists: (1) distinguishing individual cells in crowded areas, (2) distinguishing between tumor and non-tumor cells in heterogeneous areas, (3) integrating counts over a large area. Visual estimation is indeed inherently subjective and varies significantly by the examiner as reported in a widely cited study [3].

The automation of cell counting on images of H&E-stained slides using artificial intelligence (AI) is expected to not only reduce the workload of pathologists, but also to improve accuracy. AI-based methods help reduce errors by using image analysis and machine learning techniques to identify and count cells more accurately and objectively.

AI has been increasingly applied to digital pathology in recent years, with a wide range of applications in cell segmentation [4,5,6], cell counting [7,8], cell classification [9], tumor detection [10], patient outcome prediction [11,12,13,14], and image generation [15]. In particular, AI is expected to provide tools that can improve examiners’ efficacy by reducing the time they spend in tedious tasks such as identifying metastatic or mitotic cells, counting tumor cells, or searching for rare types such as signet ring cells. Additionally, AI is also expected to bring a measure of objectivity and reduce the typically large variations observed across examiners on those tasks. Finally, recent advances in deep learning models as well as the availability of fast, low-cost GPUs have pushed the boundary of what AI is capable of to the point of surpassing human accuracy [16], while keeping costs in check.

However, although AI has shown potential in a wide range of pathology at the research level, only a small number of AI-based systems are routinely used in clinical settings, especially AI systems that have been approved as medical device software [17,18].

One of the reasons why AI is not widely used in clinical pathology is that it does not adequately address the variability in image quality of input images. Specifically, after training and evaluating an AI model on a dataset collected from one site, its performance is often degraded when applied to a dataset collected from a different site.

Pathology images are affected by various factors, such as the fixation method of the pathology specimen, the thickness of the slice, the light source of the imaging device, the characteristics of the objective lens, the characteristics of the charge coupled devices (CCD) image sensor, the degree of focus at the time of scanning, as well as image postprocessing on the scanner. As a result, significant variations in color, brightness, and sharpness do occur [19]. To be truly useful in a clinical setting, AI must be robust to such variations in data source sites and imaging devices. However, it is generally difficult to collect pathology images with precise annotations from a variety of facilities using a variety of imaging devices.

Several AI approaches have been reported to address these robustness issues. One approach is to normalize the input images. This can be performed by matching the input images to the spectrum of a reference template image, or by using generative adversarial networks (GANs) to convert the input images to a target quality [20]. However, the performance of such approaches tend to deteriorate for input images that are significantly different from the template images. In addition, for GAN-based normalization, the GAN model itself also requires a large amount of computational cost to learn and may suffer from hallucinations that change the semantic content of the image, therefore introducing label-noise.

An effective method to improve the robustness of AI models from limited data is data augmentation (DA) [21,22]. This method randomly adds variations in positional information (rotation, flipping) and color, brightness, etc., to the original input images to increase the diversity of the dataset without changing the semantics of the data (location, number and type of cells). By constructing AI models from these augmented datasets, the robustness of AI to variations in image quality is improved. In [23], DA is reported to be more effective than normalization of input images for improving the robustness of AI models to staining variations.

To develop a practical AI-powered TCR calculation software, it is essential to perform cell detection and classification accurately and efficiently. Chen et al. [24] developed a method for identifying the cell cycle phase of cells based on fluorescence images. Their approach utilizes k-means clustering and rule-based heuristics to classify cells into different phases; while this method may be effective for limited datasets, its generalization performance to datasets from different institutions or acquired using different imaging devices is likely to be constrained. Ghani et al. [25] proposed a method for accelerating cell detection using a convolutional neural network embedded on an FPGA device. This approach offers significant speedups compared to software-based implementations. The authors demonstrated the effectiveness of their method in detecting different types of damaged kidney cells with an accuracy of nearly 100%. However, the limited size of their test dataset raises concerns about the generalizability of their results. Further validation with a larger and more diverse dataset is recommended.

Addressing these challenges, we leverage a previously-developed deep-learning model [26] for TCR counting and use it in this study to predict TCR for a cohort (see Section 2.1) of 41 non-small cell lung cancer patients from four different medical institutions (sites). In other reported experiments on TCR counting [27], the “gold standard” ground truth is established by first defining a tumor area and then counting all cells within that area as tumor cells. In contrast, our approach is more fine-grained and classifies each cell independently as tumor or non-tumor.

In Section 2.2, we first establish a “gold standard” (GS) set of exhaustive cell-level annotations by three pathologists in regions of interests of the cohort. We also ask 13 pathologists to visually estimate the TCR on these regions (see Section 2.3). In Section 2.4, we detail the model architecture, training partition, and data augmentation we use to create our AI model. Finally, to evaluate the real-world robustness of the AI model, we devised a leave-one-hospital-out cross-validation scheme, where we test it on images from sites unseen during training. In Section 3, we report our findings, comparing the TCR predictions by the AI model to the gold standard and to the pathologists’ visual estimate.

## 2. Materials and Methods

### 2.1. Cohort

The cohort in this study consists of 41 patient at various stages of non-small cell lung cancer (NSCLC) and considered for genetic panel testing. The specimens were collected between 2018 and 2022 from 4 sites: Hokkaido University Hospital (HUH), Kansai Medical University Hospital (KMU), Kanagawa Cancer Center (KCC), and Saitama Cancer Center (SCC). A total of 11 specimens were extracted with trans-bronchial biopsy (TBB), 9 were extracted with trans-bronchial needle aspiration (TBNA), 9 were extracted with core-needle biopsy (CNB), and 12 were extracted with surgical resections. These specimens were then prepared into blocks using the formalin-fixed paraffin-embedded (FFPE) method, sectioned, stained with hematoxylin and eosin (H&E), and scanned in bright-field at 40× magnification (0.25 microns/pixel) with a whole slide device (Philips UFS with Philips IntelliSite Pathology Solution, Philips, Amsterdam, The Netherlands) to generate WSI images.

Immunostained images from the same blocks were used during case selection for histological type differentiation but were not subsequently used for TCR estimation by pathologists nor by AI. Specimens with a high number of crushed necrotic cells were excluded. Specimen in the cohort exhibited adenocarcinoma (27) and squamous cell carcinoma (14). Care was taken that both cancer subtypes and sample extraction methods were split among sites to avoid site biases.

### 2.2. Gold Standard Labels

**Figure 1:**
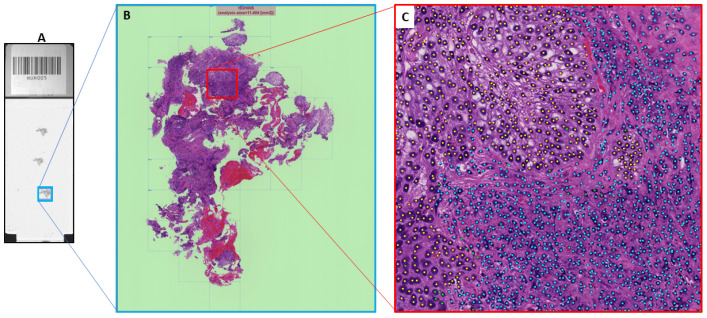
Region of interest (ROI) selection over WSI slide. In (**A**), a tissue is selected on the WSI slide. In (**B**), the tissue is tiled and one tile is selected as ROI. In (**C**), the cells of the ROI are exhaustively labeled.

In a first step, we define, for each WSI slide a region of interest (ROI) where the labeling will take place (Figure 1). To eliminate bias in selecting the ROIs from the WSI slides, we employed the following procedure:

1. A pathologist manually traces a target area on a WSI slide (typically, the tumor area).
2. A program divides the area into square tiles of 400 microns (1760 × 1760 pixels at 40× magnification) and randomly selects a tile.
3. The pathologist examines the selected tile. If it does not contain tumor cells or has an image quality issue (e.g., blurriness, artifacts), it is excluded, and another tile is randomly selected by the program. If the selected tile has none of the aforementioned issues, it is finalized as the representative ROI for the WSI.

In this manner, a representative ROI was selected from each WSI of the 41 cases, resulting in a total of 41 ROIs.

Three pathologists were then independently instructed to exhaustively identify the location of every cell in the ROI and to label them as either tumor cells (tCells), non-tumor cells (nCells), or indistinguishable cells (iCells). iCells are cells within an ROI that cannot be definitively classified as either tCells, nCells, or cells that should not be labeled. These cells may also be excluded from tumor cell content calculations by pathologists if DNA nucleic acids cannot be extracted due to crush, necrosis, degeneration, or keratinization.

In addition, cells that are not used for tumor cell content calculations by pathologists, such as red blood cells and cells whose nuclei cannot be recognized due to necrosis or degeneration, are excluded from the labeling process.

For each cell, the labels given by the three pathologists were combined into a single final label using a majority rule. Cells are initially matched using a distance-based matching algorithm thus resulting in a 3-cell match (all 3 pathologists annotated that cell), a 2-cell match, or a 1-cell match. Then, the final label was established following the rules shown in Table 1.

The annotations provided by the three pathologist labelers were then aggregated into a single final set of labels using a distance-based algorithm:

1. For each cell on the ROI, the labels from the three pathologists are gathered. Starting with a labeled cell from one labeler, the cell closest in distance from each other labeler is selected. Cells which are further than 4 microns are discarded. The resulting set of one, two or three cells are considered in the next step.
2. The final label for the set is established following the rules shown in Table 1.

In total, for the 41 ROIs, we collected approximately 40K cell labels. From those cell-level labels, the gold-standard TCR can be computed as the ratio of tCells over all cells, ignoring iCells.

(1) $$TCR_{\mathrm{GS}}=\frac{tCells_{\mathrm{GS}}}{\left(tCells_{\mathrm{GS}}+nCells_{\mathrm{GS}}\right)}$$

### 2.3. Estimate of Tumor Content Ratio

Before annotating the cells of the gold standard ROIs, 13 pathologists, including the three pathologists who created the gold standard labels, participated in a visual assessment of TCR. There was no time limit for the visual assessment, and iCells were instructed to be excluded from the estimation. Estimates were given in % with increments of 5%. Hence, for each of the 41 ROIs, we collected 13 pathologist estimates of the TCR.

### 2.4. AI Model

**Figure 2:**
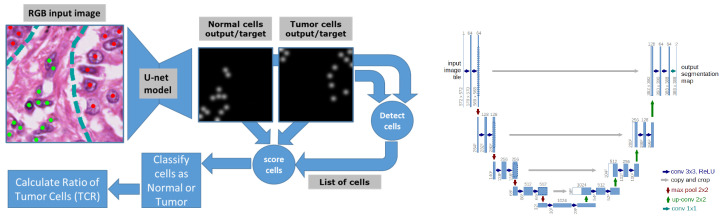
The left panel shows the overall workflow of the TCR system. An RGB image patch is the input to the model, which outputs two density maps. Annotations marking the centroid of cells (also shown as green and red overlay dots on the input image) are used to generate the two target density maps (by drawing Gaussian peaks at the location of annotated cells) which are used to train the model by back-propagating the difference (loss) between them and the maps generated by the model. The right panel shows a graphical representation of the architecture of the U-Net fully convolutional model. The numbers illustrate a 572 × 572 input image. For an 800 × 800 input image and two output maps, the total number of parameters is 28,942,850 and the total size of the model for inference (not counting training gradients) is about 3 GB.

We developed an efficient and accurate AI model capable of processing an entire WSI slide in about a minute, counting several thousand cells per second. The model is based on the fully convolutional deep learning approach and uses the U-Net neural network architecture [28]. We have described such a model in a previous publication [26], and here we only highlight the main differences with that implementation. In particular, instead of using a multi-scale approach, we use an intermediate magnification (15×) that offers a good compromise between the ability to distinguish individual cells in densely packed areas and a sufficiently large field of view (300×300 pixels at 15× magnification or 200×200 microns) that provides enough context for the model to make an educated classification of normal and tumor cells. In another departure, our memory-compact U-Net model predicts simultaneously two density maps, one for normal cells and one for tumor cells, from which a simple peak detector with a cutoff threshold and max function can efficiently return two lists of coordinates. Our approach is memory-efficient on the GPU (compact model) and minimizes CPU post-processing, allowing an entire WSI with a tissue sample of 20×20 mm to be processed under 2 minutes on a single 12 GB GPU. Figure 2 (left) illustrates the overall system and Figure 2 (right) shows the neural network layered architecture of our model.

### 2.5. AI Model Training and Evaluation Data

To establish that our model is robust to data samples collected from different hospital sites, we follow a 4-way hospital-wise cross-validation protocol. We train a model with data collected from three hospitals and test it on the data from remaining fourth hospital. The test cohort of 41 patients described in Section 2.1 provides the test data and part of the training data. Additional training data from three of the same four hospitals are used to enrich the training set. Those data are from distinct patients and are annotated by a distinct pathologist annotator following the same procedure as the test set. Those data consist of 131 ROIs from 34 patients from HUH (labeled HUNEC in Table 2), 16 ROIs from seven patients from KCC, and 3 ROIs from one patient from KMU. The 131 ROIs from HUH were collected for our initial study [26] and consist of TBB and TBNA tissue samples. Table 2 shows the 4 partitions of the data used to train and test each model. Each of the 4 partitions include training, validation, and testing partitions. We note that the training data of partition 2 technically contain HUH samples in both training (HUNEC) and test sets; however, these sets are completely different and were collected more than a year apart. Hence, we deemed it was sufficiently distinct as to not disqualify partition 2.

The scanner used for all the training data is a Nanozoomer (Hamamatsu Photonics, Hamamatsu, Japan). Note that the cohort of 41 cases has been scanned with both Nanozoomer and Philips UFS (Philips, Amsterdam, The Netherlands) scanners, with the Nanozoomer-scanned images used for with training and the Philips-scanned images used for testing. In total, the number of annotated cells for training was 212,180, roughly balanced between tumor and non-tumor cells.

### 2.6. AI Model Data Augmentation

**Figure 3:**
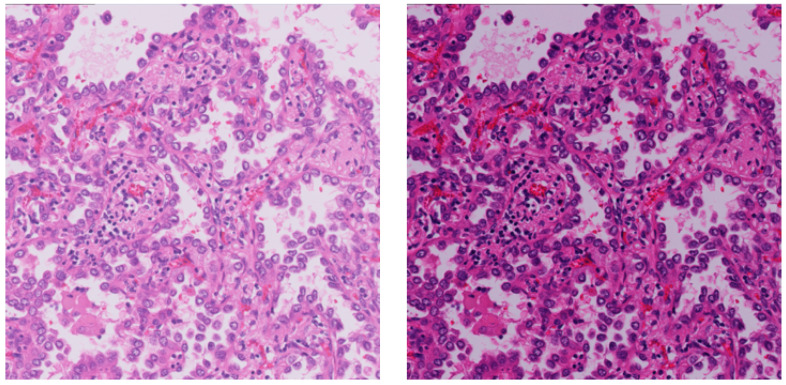
Example of data augmentation. The left image is the original, while the right image is generated by applying a gamma correction of 2.4.

Since the model is trained from images coming from a limited number of only 4 hospitals and scanned on only one model of WSI scanner (Hamamatsu Nanozoomer), we deemed that it would not be robust enough to variations encountered in samples from unseen institutions or scanners devices. Indeed, deep-learning neural-networks have a strong tendency to over-learn biases found in particular datasets. For example, image corrections applied by scanner devices for aesthetic purposes may move an image outside of the range that a model has seen during training and result in significant degradation in accuracy. Similarly, an institution may use H&E staining chemicals from different manufacturers in different quantities, resulting in variations in colors and intensity that may also fall outside the range that was seen during training. To counter this issue, data augmentation has been used almost ever since neural networks were invented. Data augmentation consists of creating new training examples automatically from existing examples. The new created images preserve the semantics of the originals and annotations obtained on the originals can still be used with the created images to train the model. Here, we simulate variations in scanner light and focusing as well as variations in staining protocols. To simulate shifts in the stain intensity of H&E, we apply color deconvolution in the optical density color space and random shift along the H (hematoxylin stain) and E (eosin stain) basis (see Figure 3). To simulate color shifts due to the scanner light, we apply intensity random shifts in the RGB color space. To simulate variations in scanner optics and the focusing mechanism, we apply random small amounts of pixel blur and sharpening. Finally, to simulate software image processing by the scanner, we apply gamma correction, brightness, contrast, and saturation shifts. We summarize the augmentations and their respective range of parameters in Table 3.

### 2.7. AI Model Training

During training, patches of image pixels (300 × 300) are extracted at random locations from the training ROIs, a random data augmentation is applied to the patch, as described in Section 2.6, and the patch is presented to the model. The model’s output is compared to the target maps generated from the annotations (by drawing Gaussian peaks at the location of annotated cells into the map corresponding to the annotation label—tumor or non-tumor) and a loss is calculated and backpropagated to update the model’s weights. The binary cross-entropy loss combined with a sigmoid layer σ(x) is used:

(2) $$L_{BCE}=-\frac{1}{N}\sum_{n}^{N}\left[y_{n}\ln\sigma \left(x_{n}\right)+\left(1-y_{n}\right)\ln\left(1-\sigma \left(x_{n}\right)\right)\right]$$

where *N* is the number of pixels in the output map, $x_{n}$ an output pixel, and $y_{n}$ a target pixel.

**Figure 4:**
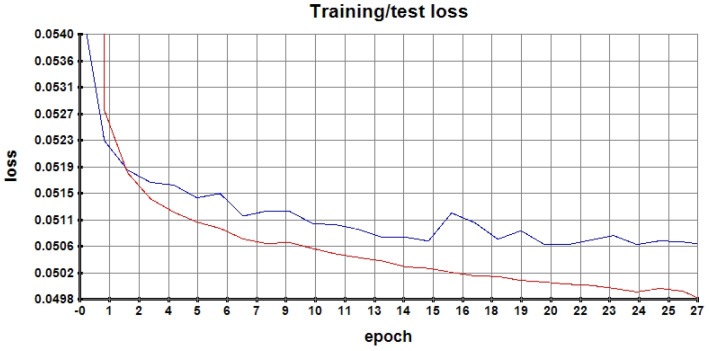
The training curve shows the cross-entropy loss as a function of the epoch. The training loss is shown in red, and the blue curve shows the validation loss.

Batch normalization is used at each layer of the model to provide a smooth learning curve. To avoid overfitting the model on the training set, training is stopped when the loss on the validation set (see partition on Table 2) stops to decrease for 4 epochs. At each epoch, the model is trained with 4000 examples and achieves convergence in about 50 epochs. Figure 4 shows the behavior of the loss during the first 27 epochs.

We use the PyTorch (version 1.10.2+cu113) [29] toolchain to train our models using the Adam [30] optimizer and a learning rate of $1\times 10^{-4}$. Training a model takes about 3 hours on a NVIDIA RTX3090 GPU.

### 2.8. AI Model Tuning of Detection Threshold and Evaluation Protocol

On the validation set, we then search for the optimal threshold for detecting cells on the output maps produced by the trained models using sequential domain reduction. A detection map is first obtained by combining the ’normal’ and ’tumor’ maps with the max operator. Peaks are then detected on the detection map when a pixel has a higher intensity than its eight neighbors and peaks with values below the detection threshold are discarded. To evaluate the accuracy of the model, we match detections with annotation labels using bipartite matching of cells within 4 microns of each other and obtain the F1-score. We then search for the threshold that maximizes the F1-score. Applying the four models with their respective detection thresholds to the test set yielded an average detection-only accuracy of 92% (balanced F1-score).

**Figure 5:**
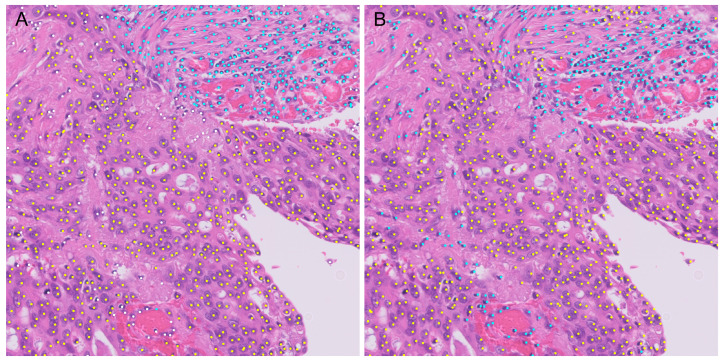
Example of tumor detection using AI. (**A**) Gold standard label. Yellow indicates tumor cells, cyan indicates non-tumor cells, and green indicates indistinguishable cells. (**B**) Predictions by the AI model. Yellow indicates tumor cells and cyan indicates non-tumor cells.

For each detected cells, a class is assigned based on the softmax of the values from the original tumor and non-tumor density maps. On the test set of 41 ROIs, we report the cell-level raw confusion matrix of the combined four leave-one-hospital-out cross-validation classification results on Table 4. We then evaluate the cell-level classification accuracy from the confusion matrix and report it on Table 5. On the same table, we also report on the third row the per-ROI classification accuracy of matched cells. Figure 5 shows an example of the output of the AI model.

Finally, we define the predicted TCR by the AI model as:

(3) $$TCR_{\mathrm{AI}}=\frac{tCells_{\mathrm{AI}}}{\left(tCells_{\mathrm{AI}}+nCells_{\mathrm{AI}}\right)}$$

where $tCells_{\mathrm{AI}}$ is the number of cells predicted by AI as tumor and matched to annotated tCells, and $nCells_{\mathrm{AI}}$ is the number of cells predicted as non-tumor by AI and matched to annotated nCells. In the calculation of $TCR_{\mathrm{AI}}$, we discarded predictions that were matched to iCells as a way to fairly compare the AI TCR with that of the pathologists’ visual estimates.

## 3. Results

We evaluated both the absolute errors of visual estimations by pathologists and of AI predictions to the gold standard TCR using the 41 annotated test cases’ ROIs. The gold standard TCR values ranged from a minimum of 1.71% to a maximum of 100%, with a mean of 62.0 ± 28.6%. The proportion of iCells (the number of iCells relative to the total number of labeled cells) ranged from 0.5% to 48.0% (mean: 17.5 ± 6.5% ).

**Figure 6:**
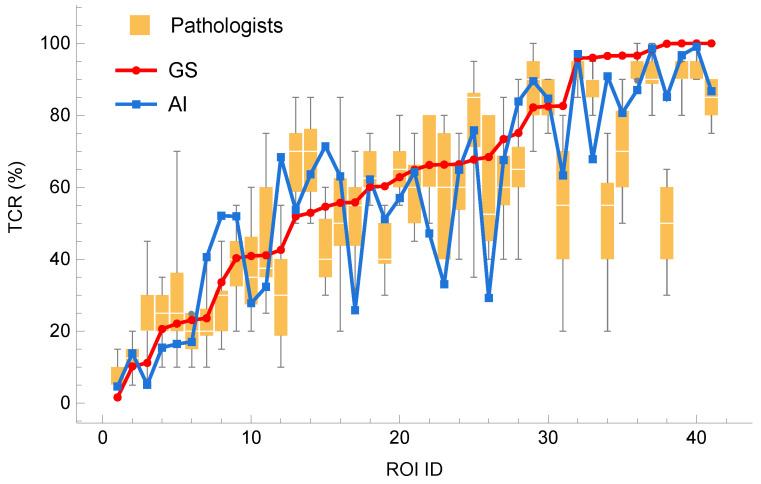
Box and whisker plot of tumor content ratio (TCR) estimated visually by 13 pathologists (yellow) together with predicted TCR by AI (solid blue line). The red solid line represent the gold standard TCR. Boxes show medians, quartiles, and outliers for each group. The boxes extend from the 25th to the 75th percentile, and the whiskers extend from the minimum to the maximum TCR within a group.

In Figure 6, we can appreciate the wide variation in the visual TCR values measured by pathologists (the largest variation is 65 %). In contrast, the largest difference between the AI’s TCR ($TCR_{\mathrm{AI}}$) and the gold standard TCR ($TCR_{\mathrm{GS}}$) is only 40%, with two cases with a difference of more than 30%. We also notice that, for both the pathologists’ visual estimate and the AI, a few ROIs with a large error strongly affect the mean error.

The mean absolute error of pathologists is defined as:

(4) $$\varepsilon_{\mathrm{PV}}=\frac{1}{N_{\mathrm{ROI}}}\sum_{i=1}^{N_{\mathrm{ROI}}}\sum_{j=1}^{N_{P}\left(i\right)}\left|TCR_{\mathrm{PV}}^{j}\left(i\right)-TCR_{\mathrm{GS}}\left(i\right)\right|,$$

where $TCR_{\mathrm{PV}}^{j}\left(i\right)$ is the TCR visually estimated by the *j*-th pathologist for the *i*-th ROI, $TCR_{GS}\left(i\right)$ is the gold standard TCR for the *i*-th ROI, $N_{ROI}$ is the number of ROIs, and $N_{P}\left(i\right)$ represents the number of pathologists who performed visual estimation for the *i*-th ROI (which varies depending on the ROI, as some pathologists were unable to participate in the visual estimation experiment for data obtained from a site). The absolute error of AI is defined as follows:

(5) $$\varepsilon_{\mathrm{AI}}=\frac{1}{N_{\mathrm{ROI}}}\sum_{i=1}^{N_{\mathrm{ROI}}}\left|TCR_{\mathrm{AI}}\left(i\right)-TCR_{\mathrm{GS}}\left(i\right)\right|,$$

where $TCR_{\mathrm{AI}}\left(i\right)$ is TCR calculated from AI predictions for the *i*-th ROI.

**Figure 7:**
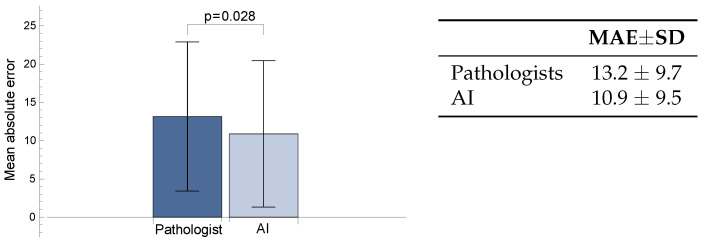
Comparison of mean absolute errors of pathologist and AI predictions to gold standard TCR.

The mean absolute errors (MAEs) of the AI-predicted and pathologist-estimated TCRs for the 41 ROIs were compared against the gold standard TCR. The AI had a mean absolute error of 10.9 ± 9.5, while the pathologist had a mean absolute error of 13.2 ± 9.7. The AI’s predictions were significantly closer to the GS TCR than the pathologist’s estimates (*p* = 0.028, with signed rank test, see Figure 7).

**Figure 8:**
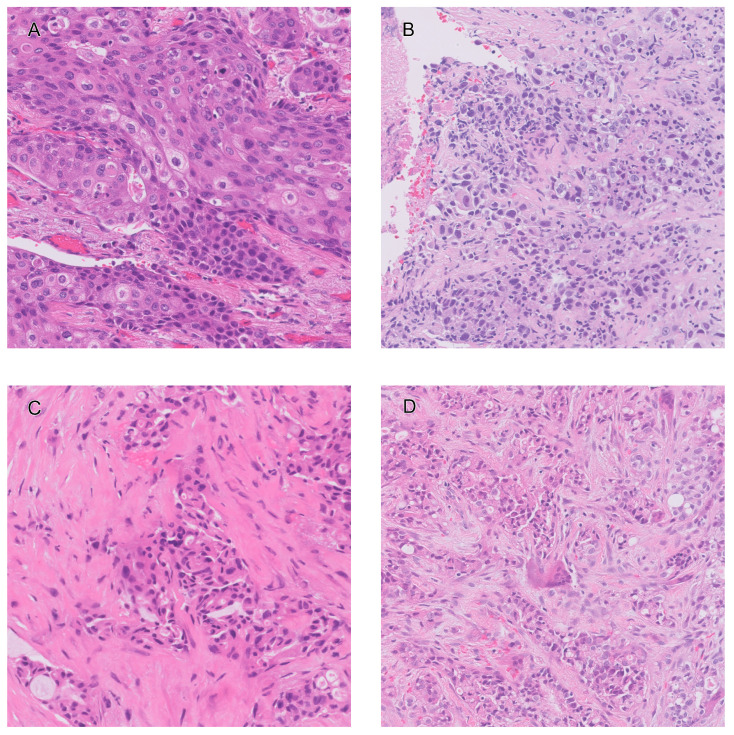
Examples of ROIs with small and large errors of pathologist’s estimation (**A**,**B**) and AI’s prediction (**C**,**D**). Pathologists’ estimations ranged from 75 to 90% for $TCR_{GS}=82.5\%$ with a mean absolute error of 5.0% (**A**), and ranged from 20 to 80% for $TCR_{GS}=82.6\%$ with a mean absolute error of 29.3% (**B**). AI’s prediction was 64.3% for $TCR_{GS}=98.4\%$ with an absolute error of 0.2% (**C**) and 33.0% for $TCR_{GS}=66.3\%$ with an absolute error of 33.3% (**D**).

Figure 8 shows ROIs that exhibited good agreement and significant deviations from the gold standard in both pathologists’ estimations and AI predictions. Figure 8A is an ROI where pathologists’ estimations exhibited good agreement with the gold standard TCR. The pathologists’ estimations ranged from 75% to 90% for $TCR_{GS}=82.5\%$ (MAE = 5.0%). AI’s prediction for this ROI was also in good agreement ($TCR_{AI}=84.6\%$, with an absolute error of 2.1%). Figure 8B shows an ROI where pathologists’ estimations exhibited significant deviation from the gold standard. The pathologists’ estimations ranged from 20% to 80% for $TCR_{GS}=82.6\%$ (MAE = 29.3%). AI’s prediction for this ROI was $TCR_{AI}=63.3\%$ with an absolute error of 19.3%).

Figure 8C shows an ROI where AI’s prediction was in good agreement with the gold standard TCR ($TCR_{AI}=64.3\%$ and $TCR_{GS}=64.9\%$, with an absolute error of 0.6%). For this ROI, pathologists’ estimations ranged from 45% to 75%, resulted in MAE = 9.2%. Figure 8D shows an ROI where AI’s prediction exhibited significant deviation from the gold standard TCR ($TCR_{AI}=33.0\%$ and $TCR_{GS}=66.3\%$, with an absolute error of 33.3%). For this ROI, pathologists’ estimations ranged from 40% to 80%, resulted in MAE = 14.6%.

**Figure 9:**
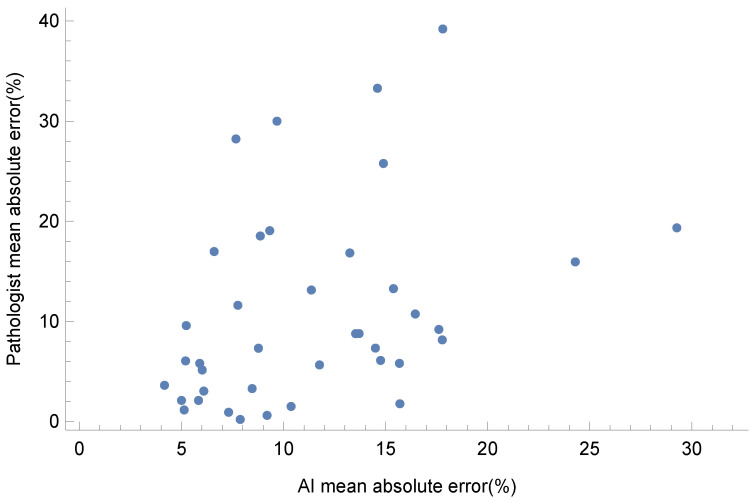
Scatter plot of the mean absolute error of the pathologist’s estimation and the AI’s prediction.

There was little overlap between the ROIs with large errors estimated by the pathologist and the ROIs with large errors calculated by the AI. In fact, as shown in Figure 9, the correlation between the absolute errors of the pathologist and the AI was small (τ = 0.265, *p* = 0.014, Kendall’s τ correlation).

### Site Dependency

**Figure 10:**
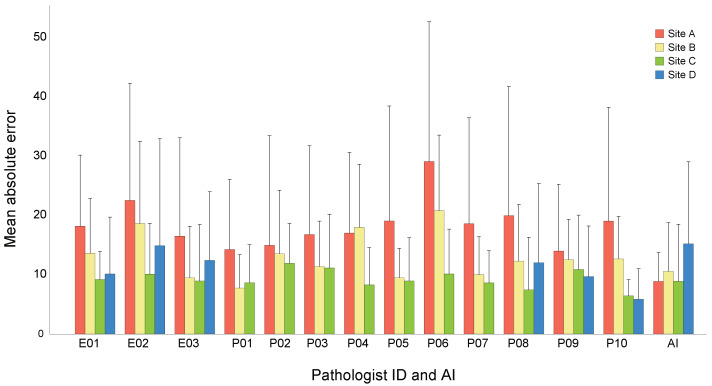
Visualization of mean absolute error of each pathologist and AI against the gold standard per site. The length of the line above the bar represents the standard deviation.

Figure 10 shows the mean absolute error for each pathologist by sites. Surprisingly, MAE varies significantly for most pathologists based on the site. To investigate this site dependence on the performance of pathologists, we define the site dependence index (SDI) as follows:

(6) $$\mathrm{SDI}={\mathrm{Max}}_{i}\left(\varepsilon_{i}\right)-{\mathrm{Min}}_{i}\left(\varepsilon_{i}\right),$$

where $\varepsilon_{i}$ is the mean absolute error within the i-th site, and ${\mathrm{Max}}_{i}$ (${\mathrm{Min}}_{i}$) is the maximum (minimum) MAE among four sites. In other words, the site dependency of each evaluator is assessed based on the difference between the site with the largest error and the smallest error. Table 6 shows the combined results of each pathologist and AI.

## 4. Discussion

In this study, we first established a gold standard tumor content ratio for a cohort of 41 cases (ROIs) from four hospitals. Three pathologists exhaustively labeled all cells on the ROIs and we used the consensus label of the three pathologists as the gold standard. We allowed the pathologists to label cells that they were not able to distinguish between tumor and non-tumor as iCells (i for indistinguishable). Since the AI model is trained to predict only between tumor and non-tumor cells, we decided to discard predictions that were matched to iCells as a way to fairly compare the AI TCR with that of the pathologists visual estimates (who were instructed to ignore them as well).

We then contrasted the accuracy of an AI model that counts and classifies every cell with that of pathologists’ visual estimation. As a result, we found that the absolute error of the AI was smaller than the mean absolute error of pathologists. This suggests that AI-based TCR measurement is useful for accurate estimation of TCR.

Figure 8 shows examples of ROIs with low and high errors for both ’pathologist visual estimation’ and ’AI counting’. In ROIs with low error, concordance was typically high between visual estimation by pathologists and AI counting. On the other hand, different trends were observed for ROIs with large errors. Visual estimation by pathologists showed large errors for tissue patterns with strong tissue degeneration and unclear nuclear borders. However, AI did not show particularly large errors for the same images. In contrast, AI showed a tendency to count small tumor cells scattered between large tumor cells as non-tumor. This is likely due to the fact that AI cannot easily identify benign-looking cells that pathologists judge to be part of a tumor nest based on a large surrounding context, as AI model makes judgments based on relatively limited input field of view of 200 square microns, encompassing only a few dozen cells. This problem could be alleviated by adopting more complex models that can consider a larger context or use attention mechanisms [31,32].

The study also evaluated the site dependency of TCR estimation error. We used the difference between the site with the largest error and the site with the smallest error as the site dependency index for each pathologist and AI. Based on this index, we found that the dependency of the pathologist was overall significantly larger than that of AI.

We observed that pathologists’ estimations were worse on site A and it was reported during post-test interviews that the staining from site A was more reddish than at other sites. Since site A was the main data collection site for the AI training, we investigated whether the AI could have been advantaged by better adapting to site A. However, such an adaptation would have resulted in significantly worse performance on other sites, which was not observed in the results.

In general, AI models are known to degrade in performance when tested on data collected from a different site than the one where the training data were collected, a phenomenon known as domain shift. Here, we observe a similar phenomenon with pathologists’ visual TCR estimation, and we show that an AI model trained with data augmentation is made robust to domain shifts encountered in the variations in sample preparation across sites. Based on these results, it is expected that the use of AI for TCR quantification will be beneficial compared to today’s pathologist visual estimation. The use of an AI model trained with data augmentation will lead to a robust TCR quantification that is resistant to differences in assessors, data collection sites (hospitals or cancer centers), and imaging devices.

The AI model used in this study is part of a software program developed by our group. The software takes a WSI file as input, detects all cells within one or more areas indicated by the user through the graphical user interface, classifies them as tumor or non-tumor cells, and reports the total TCR for all areas and as a heatmap on the WSI. In our experimental environment explained in Section 2.4, the processing time for a large surgical specimen slide of 400 mm^2^ is less than 3 min, which is very fast considering that more than a million cells are processed. Although the use of AI does not lead to a shorter examination time because the visual judgment by the pathologist is completed in a short time, it is within competitive range and has the major advantages of improved accuracy and reduced variations.

The introduction of AI to the clinical practice is not without challenges and brings fundamental changes to the workflow. Whether on-premises or in the cloud, AI computational assays will need to be competitively priced and their turnaround times will need to be clearly established. Clinical workflows will need to be adapted to use all resources optimally such that overall turnaround times from sample preparation to final diagnosis can be reduced. For example, some basic AI assays such as cell detection/classification/counting/segmentation can be systematically pre-ordered along with sample preparation and be delivered to the pathologist along with the WSI. Visualization and interpretation of those assays can happen hand-in-hand with clinical diagnosis on graphical user interface browsers and significantly increase pathologists’ productivity and accuracy.

## 5. Conclusions

The results of this study demonstrate the usefulness of AI for measuring tumor cellularity. We found that AI can calculate TCR with smaller errors than visual estimation by a pathologist by comparing both against a gold standard TCR established by exhaustively labeling each cell. In addition, the evaluation results using data obtained from different institutions showed that AI has a lower site dependency compared to the visual estimation by the pathologist. Moreover, the trend of errors between the pathologist and AI showed a small correlation, suggesting that the combined use of AI and visual estimation would be useful for high-accuracy TCR calculation.

## Figures and Tables

**Table 1:** Results of majority aggregation of three pathologists. In the first three rows, full agreement is reached, while in the bottom three rows, a tie was resolved by setting a cell to ’i’ (indistinguishable) or a cell was discarded because only one annotator labeled it. The last column indicate the number of times each situation was encountered (asterisk “*” means any label).

Matched Annotations	Agreement	Final Label	Number of Cells
3	n, n, n	n	10,040
3	t, t, t	t	14,587
3	i, i, i	i	2824
3	n, n, NOT-n	n	949
3	t, t, NOT-t	t	1047
3	i, i, NOT-i	i	1591
2	t, t	t	21
2	n, n	n	24
2	i, i	i	20
3	n, t, i	i	92
2	*, NOT-*	i	8
1	n OR t OR i	discard	93 + 78 + 132 = 303

**Table 2:** Four partitions used for the leave-one-hospital-out cross-validation. The number in parenthesis represent the number of images used for each institution and each task.

Partition	Test Set	Training Set	Validation Set
1	SCC (10)	HUNEC (75) + HUH (11) + KMU (12) + KCC (27) = 125	HUNEC (56)
2	HUH (11)	HUNEC (75) + SCC (10) + KMU (12) + KCC (27) = 124	HUNEC (56)
3	KMU (9)	HUNEC (75) + SCC (10) + KCC (27) + HUH (11) = 123	HUNEC (56)
4	KCC (11)	HUNEC (75) + SCC (10) + KMU (12) + HUH (11) = 108	HUNEC (56)
total	41	135	56

**Table 3:** Data augmentation parameters for color and blurriness/sharpness. The algorithm first randomly decides to apply either HE or RGB color shift, then randomly decides either gamma or BCS, and finally randomly decides between blur, sharpen, and nothing.

Augmentation	Parameters
Hematoxylin and Eosin (HE)	random add ±16% of range
Red, Green Blue	random add ±12% of range
Gamma	random apply Gamma correction within [0.4; 1.5]
Brightness	random subtract 15% of range
Contrast and Saturation	multiply by a random value within [70%; 130%]
Blur (Gaussian)	Convolve with Gaussian kernel sigma = random {0.3, 0.5}
Sharpen	Apply sharpen filter with random intensity in [1.0; 1.2]

**Table 4:** Cell-level confusion matrix of the combined 4 leave-one-hospital-out cross-validation classification results on 41 ROIs.

	Prediction Non-Tumor	Prediction Tumor	Unmatched Label	Total Labels
Label Non-Tumor	NN = 10,762 (26%)	NT = 2044 (5%)	ULN = 1408 (3%)	14,214 (34%)
Label Tumor	TN = 3171 (7%)	TT = 17,174 (42%)	ULT = 1644 (4%)	21,989 (53%)
Label Indistinguishable	IN = 2541 (6%)	IT = 1535 (3%)	ULI = 664 (1%)	4740 (11%)
Unmatched Predictions	UPN = 1678 (4%)	UPT = 1430 (3%)	N/A	3108 (7%)
Total Predictions	18,152 (45%)	22,183 (54%)	3716 (9%)	40,335; 40,943

**Table 5:** Cell-level sensitivity and specificity of the combined 4 leave-one-hospital-out cross-validation classification results on 41 ROIs calculated from the confusion matrix of Table 4. The first row reports the accuracies for only the predicted cells that were matched to a label: $SEN=\frac{TT}{TT\;+\;TN}$ $SPE=\frac{NN}{NN\;+\;NT}$. The second row reports accuracies that also include the predicted cells that were not matched to a label (false positives) as well as the labels that were not matched to a prediction (false negatives): $SEN=\frac{TT}{TT\;+\;TN\;+\;ULT}$ $SPE=\frac{NN}{NN\;+\;NT\;+\;ULN}$. The third row reports the per-ROI matched cells performance averaged over the 41 ROIs.

	Sensitivity	Specificity
Matched Cells Only	84.4%	84.0%
All Cells	78.1%	75.7%
ROI-level	80.8%	79.0%

**Table 6:** Site dependency indices and mean absolute error of each pathologist and AI against the gold standard per site. Data for 7 pathologists (ID = P01–P07) are missing because they did not participate in the visual estimation of cases for Site D.

Site	Pathologist														AI
	E01	E02	E03	P01	P02	P03	P04	P05	P06	P07	P08	P09	P10	Mean ± SD	
A	18.2	22.6	16.5	14.3	15.0	16.8	17.0	19.1	29.1	18.6	20.0	14.0	19.1	18.5 ± 4.0	8.9
B	13.6	18.6	9.5	7.8	13.5	11.4	18.0	9.5	20.8	10.0	12.3	12.6	12.7	13.1 ± 3.9	10.6
C	9.2	10.1	9.0	8.7	11.9	11.1	8.3	9.0	10.1	8.6	7.5	10.9	6.5	9.3 ± 1.5	8.9
D	10.2	14.9	12.4								12.0	9.7	5.9	10.6 ± 3.0	15.2
SDI	9.0	12.5	7.6	6.5	3.0	5.6	9.7	10.1	19.0	10	12.5	4.3	13.2	9.5 ± 4.7	6.3

## Data Availability

The datasets presented in this article are not readily available because the data are part of an ongoing study.

## Abbreviations

AI	artificial intelligence
NGS	next-generation sequencing
TCR	tumor content ratio
H&E	hematoxylin and eosin
GANs	generative adversarial networks
DA	data augmentation
ROI	region of interest
GS	gold standard
NSCLC	non-small cell lung cancer
TBB	trans-bronchial biopsy
TBNA	trans-bronchial needle aspiration
CNB	core-needle biopsy
FFPE	formalin-fixed paraffin-embedded
WSI	whole-slide image
tCell	tumor cell
nCell	non-tumor cell
iCell	indistinguishable cell
MAE	mean absolute errors
SDI	site dependency index
